# Ablation of ventricular tachycardia originating from a reconstructed left ventricular aneurysm: expanding the role of the lattice-tip catheter

**DOI:** 10.1093/ehjcr/ytaf573

**Published:** 2025-10-31

**Authors:** Antonios Martinos, Andromahi Zygouri, Vasileios Cheilas, Anna Kostopoulou

**Affiliations:** Arrythmia Unit and Electrophysiology Laboratory, Onassis Hospital, L. Syngrou 356, Athens 17674, Greece; Arrythmia Unit and Electrophysiology Laboratory, Onassis Hospital, L. Syngrou 356, Athens 17674, Greece; Arrythmia Unit and Electrophysiology Laboratory, Onassis Hospital, L. Syngrou 356, Athens 17674, Greece; Arrythmia Unit and Electrophysiology Laboratory, Onassis Hospital, L. Syngrou 356, Athens 17674, Greece

## Case description

An 84-year-old male presented to the emergency department with multiple episodes of sustained monomorphic ventricular tachycardia (VT) with a cycle length of 360 ms treated with unsuccessful antitachycardia pacing attempts and 11 defibrillator shocks. His medical history includes a previous non-revascularized inferior myocardial infarction, a ‘modified Dor’ aneurysm reconstruction, and an implantation of a cardiac defibrillator (ICD) 11 years prior.

**Figure 1:**
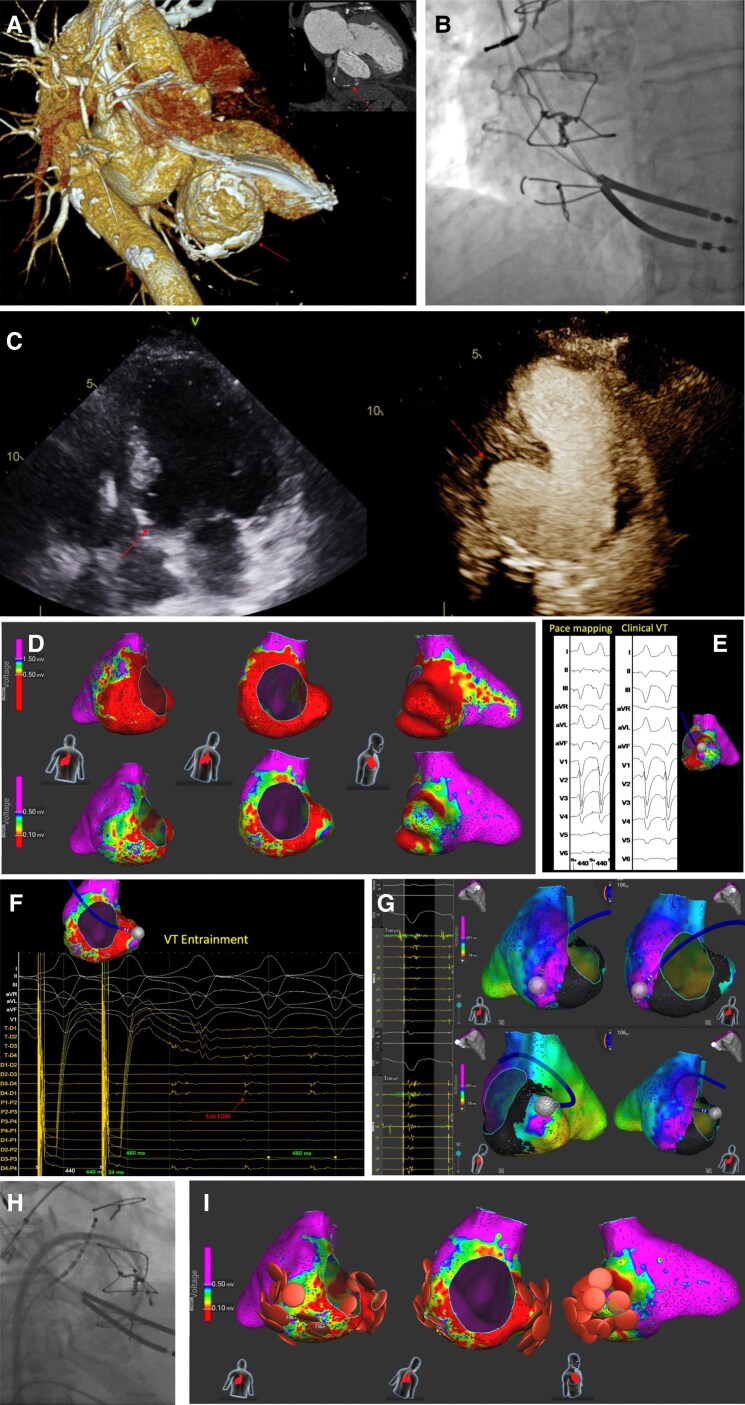
Multimodality imaging, mapping, and ablation of ventricular tachycardia originating from a left ventricular aneurysm. (*A*) Cardiac CT scan: 3D reconstruction demonstrates a calcified aneurysm involving the basal inferoseptal and basal inferior left ventricular segments (arrow). The upper right panel displays a two-chamber view, highlighting the calcified basal inferior aneurysm (dashed arrow). (*B*) Coronary angiogram: The left anterior oblique view of the right coronary artery (RCA), showing a chronic total occlusion in the mid RCA segment (pre-existing finding). (*C*) Transthoracic echocardiography. *Left panel:* apical four-chamber view, depicting an aneurysm at the basal inferoseptal wall (arrow). *Right panel:* contrast-enhanced apical two-chamber view highlighting an aneurysm at the basal inferior wall (dashed arrow). (*D*) Substrate mapping: Bipolar voltage maps of the left ventricle using a 3D electroanatomic mapping system (Affera, Medtronic). *Upper panel:* The lower bipolar voltage threshold was set to 0.5 mV, revealing extensive scar tissue (red) at the aneurysm site and adjacent inferolateral wall. *Lower panel:* The threshold was adjusted to 0.1 mV to delineate areas of dense scar and potential ventricular VT corridors. Regions depicted in purple correspond to preserved myocardium (bipolar voltage >1.5 mV in the upper panel and >0.5 mV in the lower panel). (*E*) Pace mapping: Pace mapping performed at the aneurysm site, demonstrating a good morphology match with the induced clinical VT. (*F*) Entrainment mapping: VT was entrained by pacing from the t pole of the Sphere-9 catheter, accelerating VT cycle length to 440 ms. The diastolic electrogram was captured and suppressed during pacing, returning with a post-pacing interval (PPI) equal to the tachycardia cycle length (TCL). Concealed fusion on ECG, along with a morphology matching the clinical VT and a stimulus-to-QRS interval of 34 ms identifies this site as the likely VT exit. (*G*) Late potential identification: Areas of late potentials (highlighted in purple) were detected within the basal inferoseptal aneurysm and the basal inferolateral wall, using a 3D electroanatomic mapping system (Affera, Medtronic). (*H*) Catheter positioning: Right anterior oblique view during the procedure, showing the lattice-tip focal catheter positioned in the basal inferoseptal aneurysm via an antegrade transseptal approach and a steerable sheath. (*I*) Ablation strategy: Substrate modification with radiofrequency temperature-controlled applications (30 s per lesion), targeting and eliminating the VT exit site and late potentials regions within the basal inferoseptal and inferior aneurysm walls.

The patient was on optimal medical therapy for heart failure, including metoprolol. Antiarrhythmic therapy was escalated with the addition of mexiletine and amiodarone. Echocardiography showed a large basal inferoseptal and inferior wall aneurysm (*Figure 1C*). Coronary angiography revealed chronic mid-right coronary artery occlusion (*Figure 1B*), while cardiac CT detailed the aneurysm’s size (6.02 × 3.46 mm), adhesions, and wall thickness (*Figure 1A*).

Catheter ablation was performed due to persistent arrhythmias. The 9 mm lattice-tip catheter (Sphere-9, Medtronic), integrated within the AFFERA electro-anatomical mapping, and ablation system was chosen mainly for its shape, larger footprint, and dual energy options. Using the lattice-tip catheter via an antegrade transseptal approach (*Figure 1H*), substrate maps were created during stable atrial pacing at 0.5 and 0.1 mV lower voltage thresholds to identify dense scar and potential corridors^1^ (*Figure 1D*). Fragmented and late potentials were tagged during mapping (*Figure 1G*). Pace mapping and entrainment^2^ helped localize the VT exit site (*Figure 1E* and *1F*). Activation mapping during VT was not possible because of haemodynamic instability. A total of 35 radiofrequency temperature-controlled applications (total duration 521 s, 30 s/lesion, temperature limit at 60°C) targeted the VT exit, inferoseptal border of the aneurysm, and late potential areas for scar homogenization (*Figure 1I*). VT was not inducible after ablation.

The patient had no arrhythmia at 5 month follow-up. This case aligns with findings from recent registries that support the feasibility and safety of the lattice-tip catheter for ventricular tachycardia ablation in complex substrates.^3^

## Supplementary Material

ytaf573_Supplementary_Data

## Data Availability

The data underlying this article will be shared on reasonable request to the corresponding author.
